# Supplementary material for: Confidence reports during perceptual decision making dissociate from changes in subjective experience
Source: Commun Psychol. 2025 May 21;3:81. doi: 10.1038/s44271-025-00257-y (PMC12095063; doi:10.1038/s44271-025-00257-y)
Supplement: Supplementary file 2 — Supplementary materials [file 44271_2025_257_MOESM2_ESM.pdf]

Supplementary materials: Confidence reports during perceptual decision making dissociate from changes in subjective experience

Nicolás Sánchez-Fuenzalida[1,2,3,4], Simon van Gaal[1,2], Stephen M. Fleming[5,6], Julia M. Haaf[1,7], and Johannes Jacobus Fahrenfort[3,4,1,2]

[1]Department of Psychology, University of Amsterdam
[2]Amsterdam Brain & Cognition, University of Amsterdam
[3]Department of Applied and Experimental Psychology, Free University Amsterdam
[4]Institute for Brain and Behavior Amsterdam, Free University Amsterdam
[5]Department of Experimental Psychology, University College London
[6]Max Planck Centre for Computational Psychiatry and Ageing Research, University College London
[7]Department of Psychology, University of Potsdam

Supplementary text T1: General procedure

For the line length categorization task, participants first completed 10 trials with feedback with no performance demands, then 10 correct practice trials in a row with feedback, 10 correct practice trials without feedback and finally a longer, more difficult block of 25 trials without feedback with at least 80% correct responses. After the categorization instructions, participants received instructions about how to provide confidence reports about their decision (low and high) (see supplementary figure S1 and S2 for the full confidence report instructions). Participants completed 20 confidence practice trials where half of the trials were very difficult compared with the other half of the trials. Participants were required to evenly use high and low confidence reports, so that difficult trials were more often labelled as low confidence trials, and easy trials were more often labelled as high confidence trials. Participants then received instructions for the reproduction task in the same way as described for the length categorization (decision) task. In the reproduction task a deviation greater than 40 pixels from the length of the target line was considered an error (regardless of whether it was above or below the target line length). Afterwards, participants completed 25 practice trials where both tasks (categorization and reproduction) were intermixed, just as in the actual experiment (see Supplementary figure S16 for a graphical depiction of the procedure). After the tasks' instructions and practice, participants in the payoff and base rate conditions were instructed about the asymmetrical punishment and stim-prevalence scheme just before the experimental trials started. In both the payoff and base rate conditions, participants completed an extra practice block that consisted of 25 trials with trial-by-trial feedback where either the payoff or base rate manipulation was in place to confirm they understood the instructions. In the Müller-Lyer condition participants were explicitly instructed to ignore the flanking arrowheads and to solely judge the length of the horizontal target lines. In the payoff and base rate condition a similar instruction was given about the flanking vertical lines.

During the experiment, you will sometimes be confident that the target line is longer or shorter than the reference line, while at other times you may not be so sure. Therefore, we also need you to report how confident you are in your decision. To do so, you can use a single response that indicates both your decision (longer or shorter) and the confidence you have in your decision(low confidence or high confidence), as shown in the picture below. When you are relatively confident use the Z (high-confidence shorter) or right mouse button (high-confidence longer), when you are relatively unsure, use the X (low-confidence shorter) or left mouse button (low-confidence longer). Try to use the LOW/HIGH confidence options properly throughout the experiment, so using HIGH when you are relatively confident compared to other trials, and using LOW when you are relatively 'unsure compared to other trials.

You will go over some practice trials so you can get used to the confidence/decision answer. Remember, use HIGH when you are relatively confident compared to other trials, and use LOW when you are relatively unsure compared to other trials.

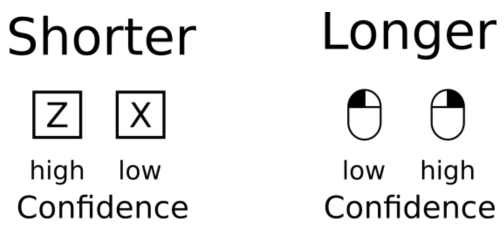

Fig. S1: Concurrent decision confidence instructions. During the discrimination instructions participants were prompted to concurrently report a confidence rating. After these instructions participants went over a practice block where they were required to evenly use “high” and “low” confidence responses.

During the experiment, you will sometimes be confident that the target line is longer or shorter than the reference line, while at other times you may not be so sure. Therefore, we also need you to report how confident you are in your decision. On every trial, after answering SHORT/LONG you will have to report the confidence you have in your decision (low confidence or high confidence), as shown in the picture below. When you are relatively confident use the 'W' key, when you are relatively unsure use the 'S' key. Try to use the LOW/HIGH confidence options properly throughout the experiment, so using HIGH when you are relatively confident compared to other trials, and using LOW when you are relatively unsure compared to other trials.

You will go over some practice trials so you get used to answering LOW/HIGH confidence after answering SHORT/LONG. Remember, use HIGH when you are relatively confident compared to other trials, and use LOW when you are relatively unsure compared to other trials.

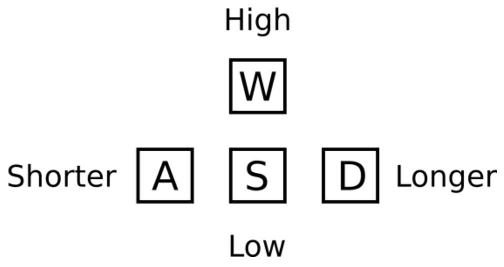

Fig. S2: Post-decision confidence instructions. During the discrimination instructions participants were prompted to concurrently report a confidence rating. After these instructions participants went over a practice block where they were required to evenly use “high” and “low” confidence responses.

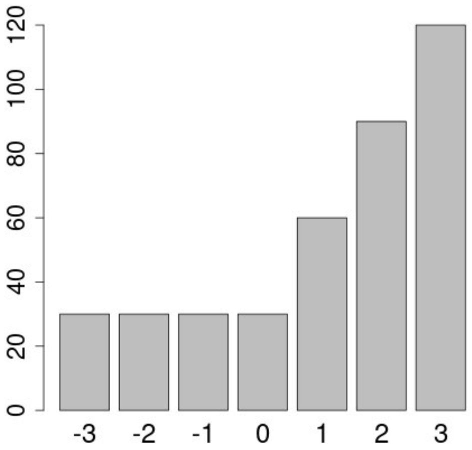

Fig. S3: Base rate frequency of each target length. Frequency of each target length value in the base rate condition biased to long (histogram was mirrored when the bias direction was ‘short’). The y-axis indicates the absolute frequency of each target length in the x-axis. In the x-axis zero represents the length of the reference line and each step away from the centre indicates an decrease/increase of 10 pixels.

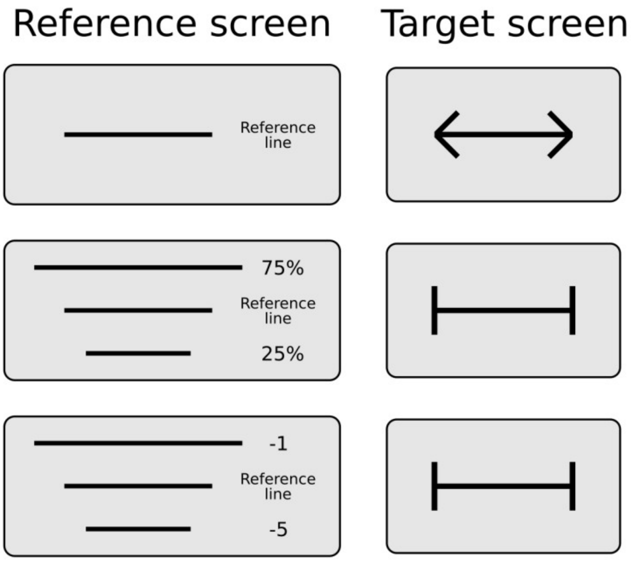

Supplementary figure S4: Reference and target screen examples. On the left column there is an example of the reference screen of the Müller-Lyer (top row), base rate biased to long (middle row) and payoff biased to long (bottom row) conditions. In the Müller-Lyer condition the reference screen looked the same for biased to long and short conditions. In the base rate and payoff conditions the numbers indicating the relative frequency of short and long lines, or the cost for incorrectly answering ‘short’ or ‘long’ was inverted depending on the bias direction. On the right column there is an example of the target screen for the same conditions in the same order as for the left column. In the Müller-Lyer condition the arrowheads pointed outwards when the bias direction was short or inward when the bias direction was long. In the base rate and payoff condition the target lines were always flanked by vertical lines, regardless of the bias direction.

You have completed 48% of the experiment.
You are doing well!

You incorrectly answered LONG 6 times (-5 each), you lost 30 points.
You incorrectly answered SHORT 2 times (-2 each), you lost 2 points.
Your length reproductions were too off-track 7 times (-3 each), you lost 21 points.

In the previous block, you indicated LOW confidence on 43% of the trials and HIGH confidence on 57% of the trials.

You have completed 48% of the experiment.
You are doing well!

You made 6 mistakes (-3 each), you lost 18 points.
Your length reproductions were too off-track 7 times (-3 each), you lost 21 points.

In the previous block, you indicated LOW confidence on 14% of the trials and HIGH confidence on 86% of the trials.

Remember that your response should reflect your relative confidence compared to the other trials. When you do this, you should have a similar percentage for LOW and for HIGH confidence responses at the end of every block. Try to use the LOW and HIGH confidence response more evenly.

Fig. S5: Block-level feedback example. Examples of the feedback participants received at the end of each experimental block in the payoff condition (top panel) and in the Müller-Lyer condition (bottom panel). In the base rate condition participants received the same message as in the payoff condition with the exception that the cost for incorrect “long” and “short” responses was the same (-3 points). In all conditions participants also received feedback on the relative use of “high” and “low” confidence responses. When participants reported “high” or “low” confidence in less than 25% of the trials of a block they were prompted to try to use both “high” or “low” as evenly as possible (see bottom panel).

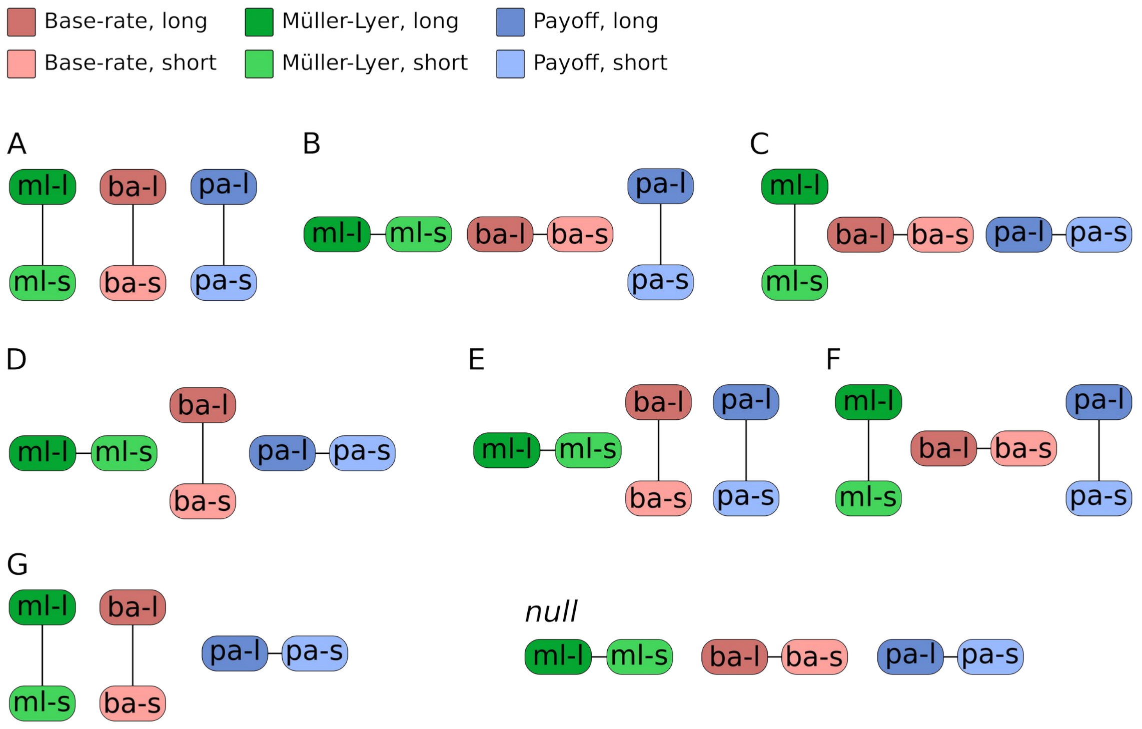

Fig. S6: Bayesian ordinal models. Each cell refers to the mean PSE (decision task), peak uncertainty (confidence task) or reference length reproduction (reproduction task). On each cell 'ba' refers to base rate, 'ml' to Müller-Lyer and 'pa' to payoff. While 'l' refers to biased to long and 's' biased to short. Vertical lines connecting cells indicate that the top cell has a higher value than the bottom cell (non-zero positive effect size), while horizontal lines between cells indicate null-effects. The effects across bias source conditions are not constrained, meaning that the models only specify whether the effect between bias-to-long and bias-to-short within a given bias source is zero or non-zero.

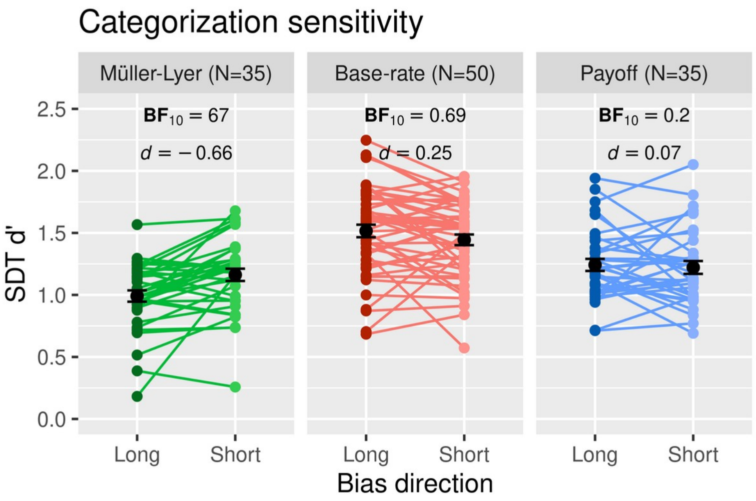

Fig. S7: Experiment 1: Categorization sensitivity. SDT d' values for each subject are plotted along with the group average for each bias source and bias direction condition. All error bars are standard errors of the mean. Higher values indicate a better performance at the task. BF values correspond to a two-sided Bayesian t-test with a default Cauchy prior of 0.707. *d* values correspond to Cohen's d effect size coefficients.

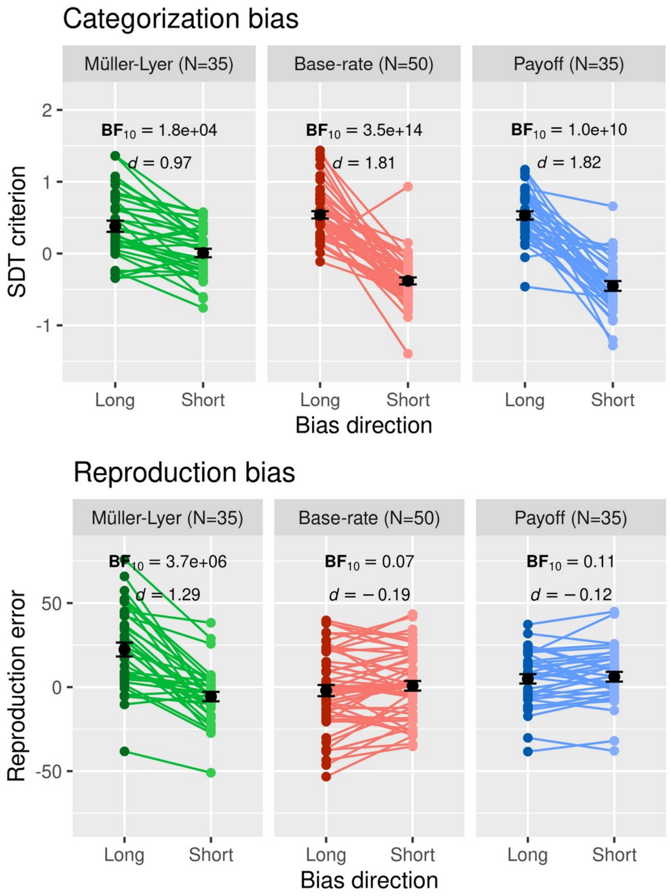

Fig. S8: Experiment 1. Decision bias and reproduction results. A) Categorization bias. The SDT criterion value for each subject along with the group average for each bias source and bias direction condition. Higher values indicate a stronger bias towards answering ‘long’ while lower values indicate a stronger bias towards answering ‘short’. **B) Reproduction error.** The average reproduction error (*length reproduction - target length*) for each subject is displayed for each bias source and bias direction condition. Higher values indicate lines reproduction longer than the target line while lower values indicate lines reproduction shorter than the target line. All error bars are standard errors of the mean. BF values correspond to a one-sided Bayesian t-test with a default Cauchy prior of 0.707. *d* values correspond to Cohen’s d effect size coefficients.

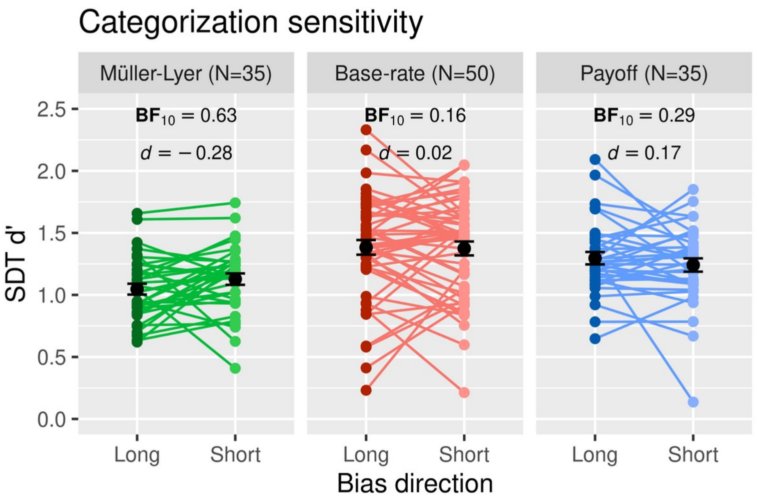

Fig. S9: Experiment 2: Categorization sensitivity. SDT d' values for each subject are plotted along with the group average for each bias source and bias direction condition. All error bars are standard errors of the mean. Higher values indicate a better performance at the task. BF values correspond to a two-sided Bayesian t-test with a default Cauchy prior of 0.707. d values correspond to Cohen's d effect size coefficients.

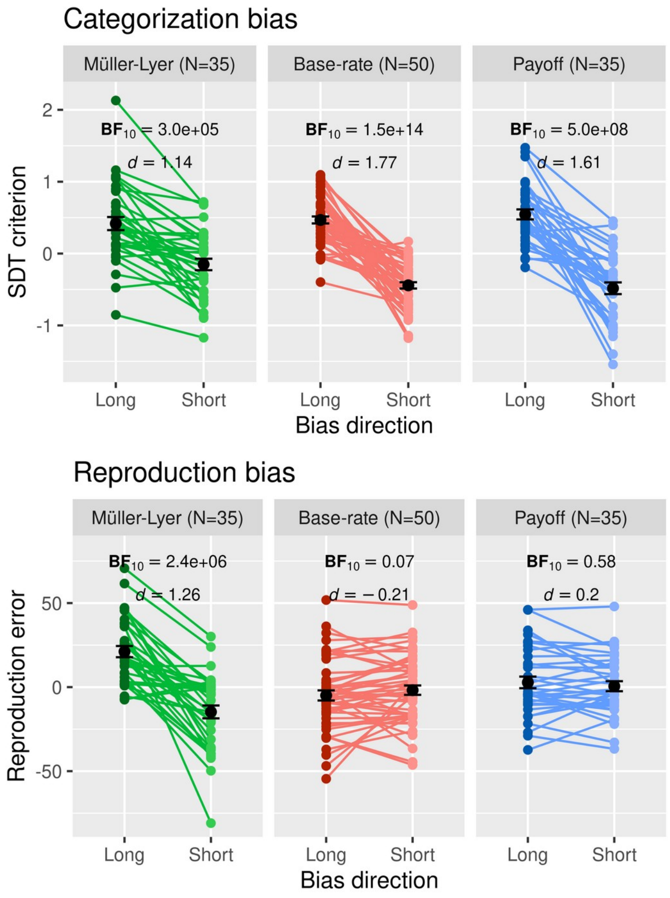

Fig. S10: Experiment 2. Decision bias and reproduction results. A) Categorization bias. The SDT criterion value for each subject along with the group average for each bias source and bias direction condition. Higher values indicate a stronger bias towards answering ‘long’ while lower values indicate a stronger bias towards answering ‘short’. **B) Reproduction bias.** The average reproduction error (*length reproduction - target length*) for each subject is displayed for each bias source and bias direction condition. Higher values indicate lines reproduction longer than the target line while lower values indicate lines reproduction shorter than the target line. All error bars are standard errors of the mean. BF values correspond to a one-sided Bayesian t-test with a default Cauchy prior of 0.707. d values correspond to Cohen’s d effect size coefficients.

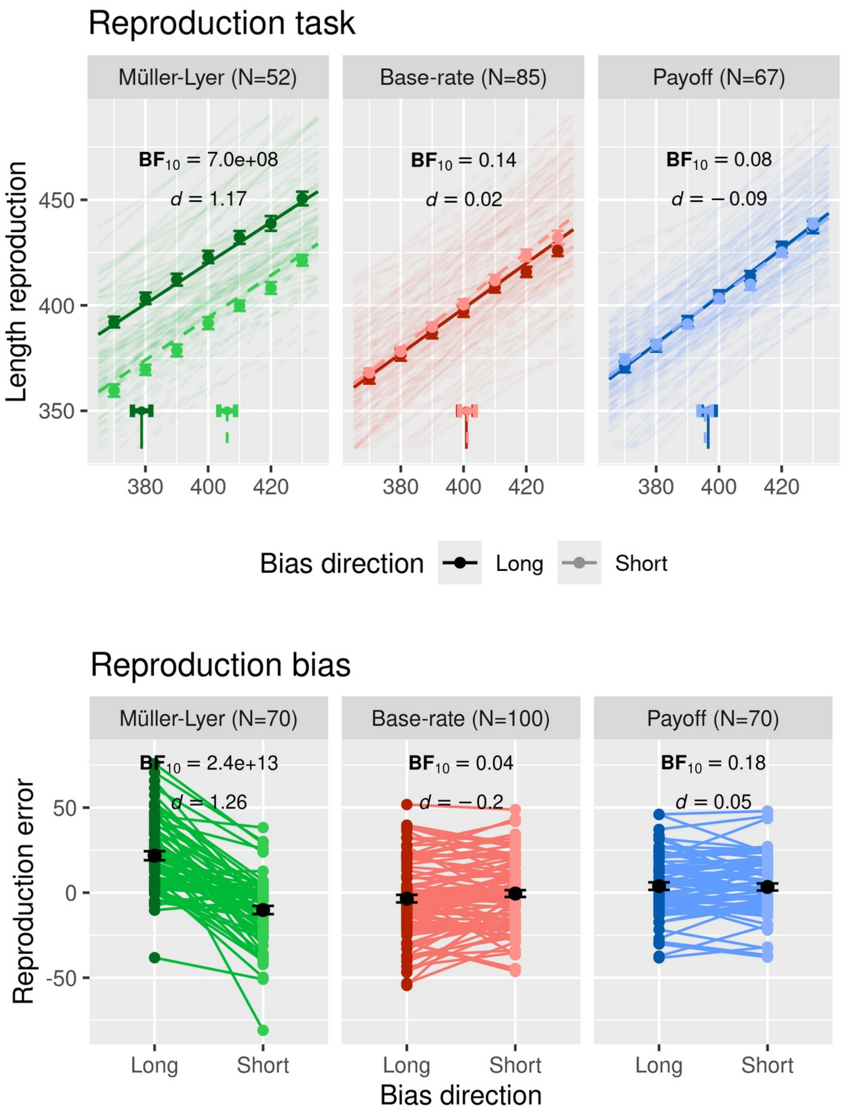

Supplementary figure S11: Experiment 1 and 2 combined reproduction data. Top panel: A straight line was fitted to each observer's distribution of length reproductions of experiment 1 and 2. At the bottom of each panel is plotted the target length associated with reproductions equal to the reference line (400 pixels). Points plotted over the fitted line correspond to the average reproduced length for each target line presented. **Bottom panel:** The average reproduction error (*length reproduction - target length*) for each subject is displayed for each bias source and bias direction condition. Higher values indicate lines reproduction longer than the target line while lower values indicate lines reproduction shorter than the target line. All error bars are standard errors of the mean. BF values correspond to a one-sided Bayesian t-test with a default Cauchy prior of 0.707. *d* values correspond to Cohen's d effect size coefficients.

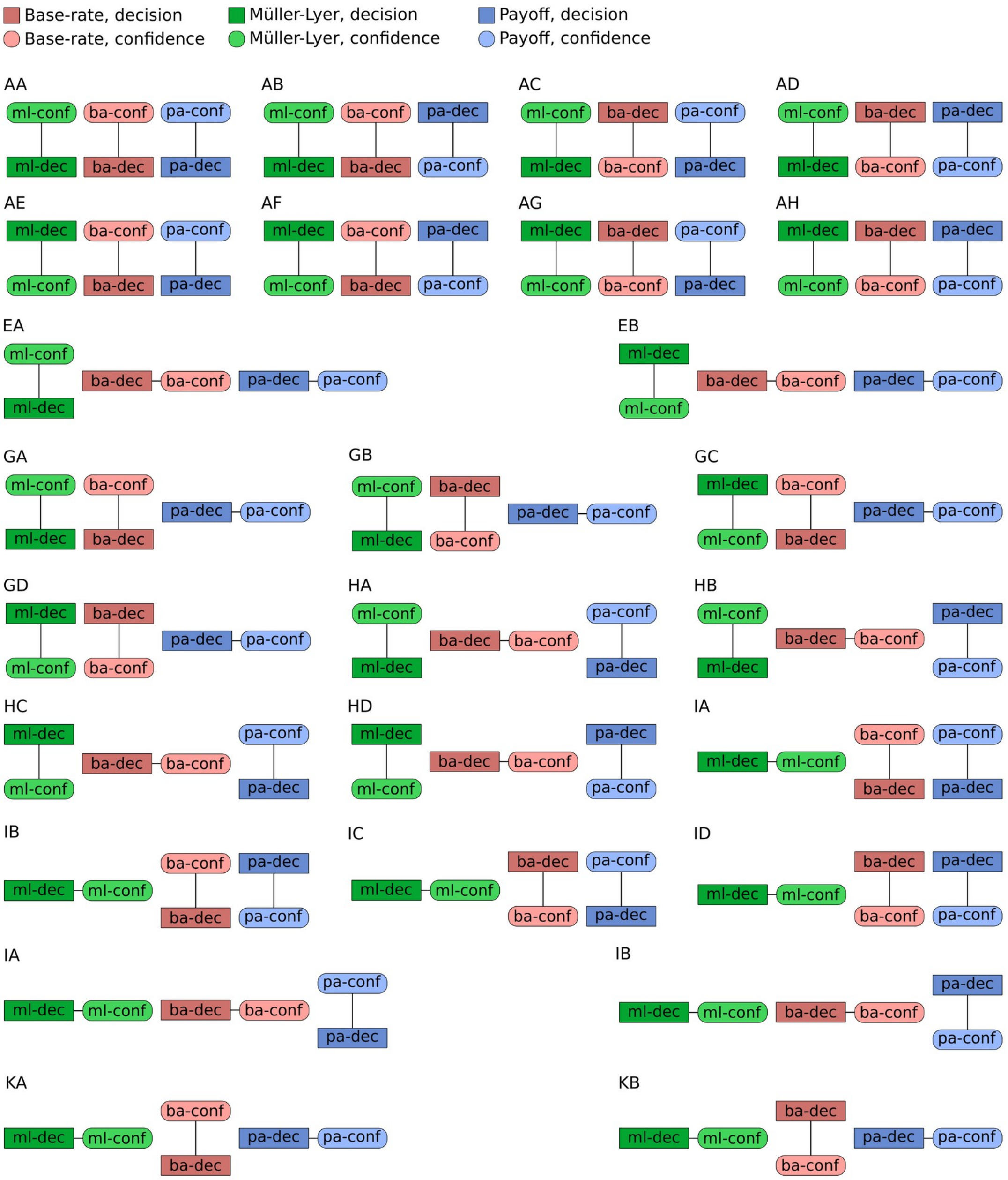

Fig. S12: Bayesian ordinal models for decision and confidence effects. Each cell refers to the mean effect on the decision task (PSE difference between the biased-to-long and biased-to-short conditions) or the mean effect on the confidence task (PMU difference between the biased-to-long and biased-to-short conditions). On each cell ‘ba’ refers to base rate, ‘ml’ to Müller-Lyer and ‘pa’ to payoff. While ‘dec’ refers to the decision task and ‘conf’ to the confidence task. Vertical lines connecting cells indicate that the top cell has a higher value than the bottom cell (non-zero positive effect size), while horizontal lines between cells indicate null-effects. The effects across bias source conditions are not constrained, meaning that the models only specify whether the effect on the decision task and the confidence task within a given bias source are zero or non-zero.

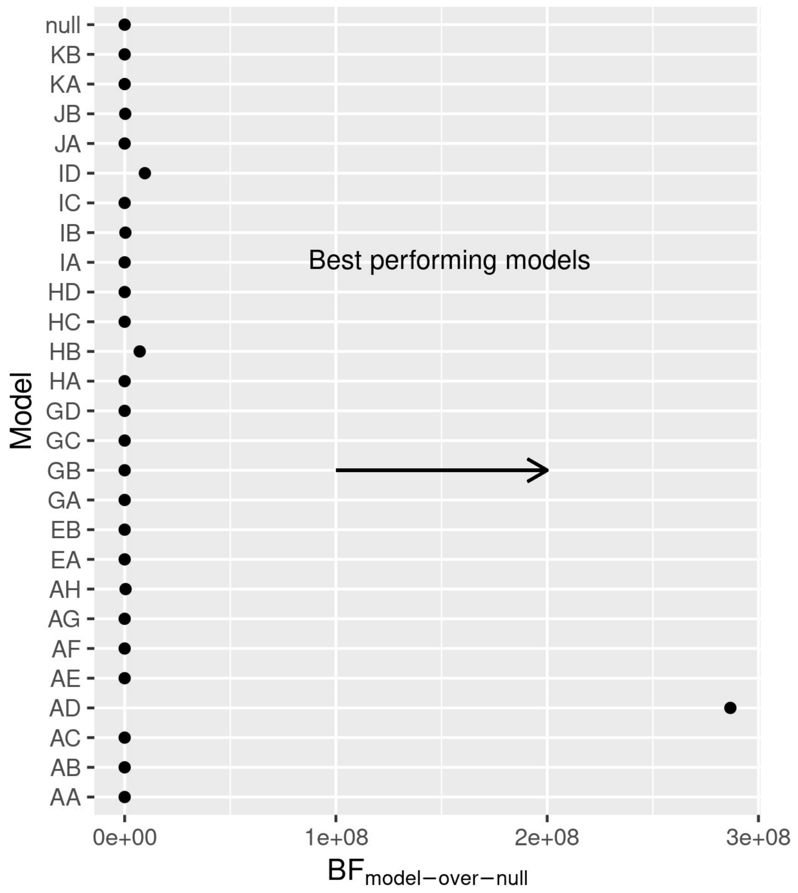

Figure S13: BF values of ordinal models tested on the effects of the decision and confidence task. The BF value of each model on the y-axis over the null model. Higher values indicate better performing models. See Supplementary Figure S12 for a graphical depiction of the models tested.

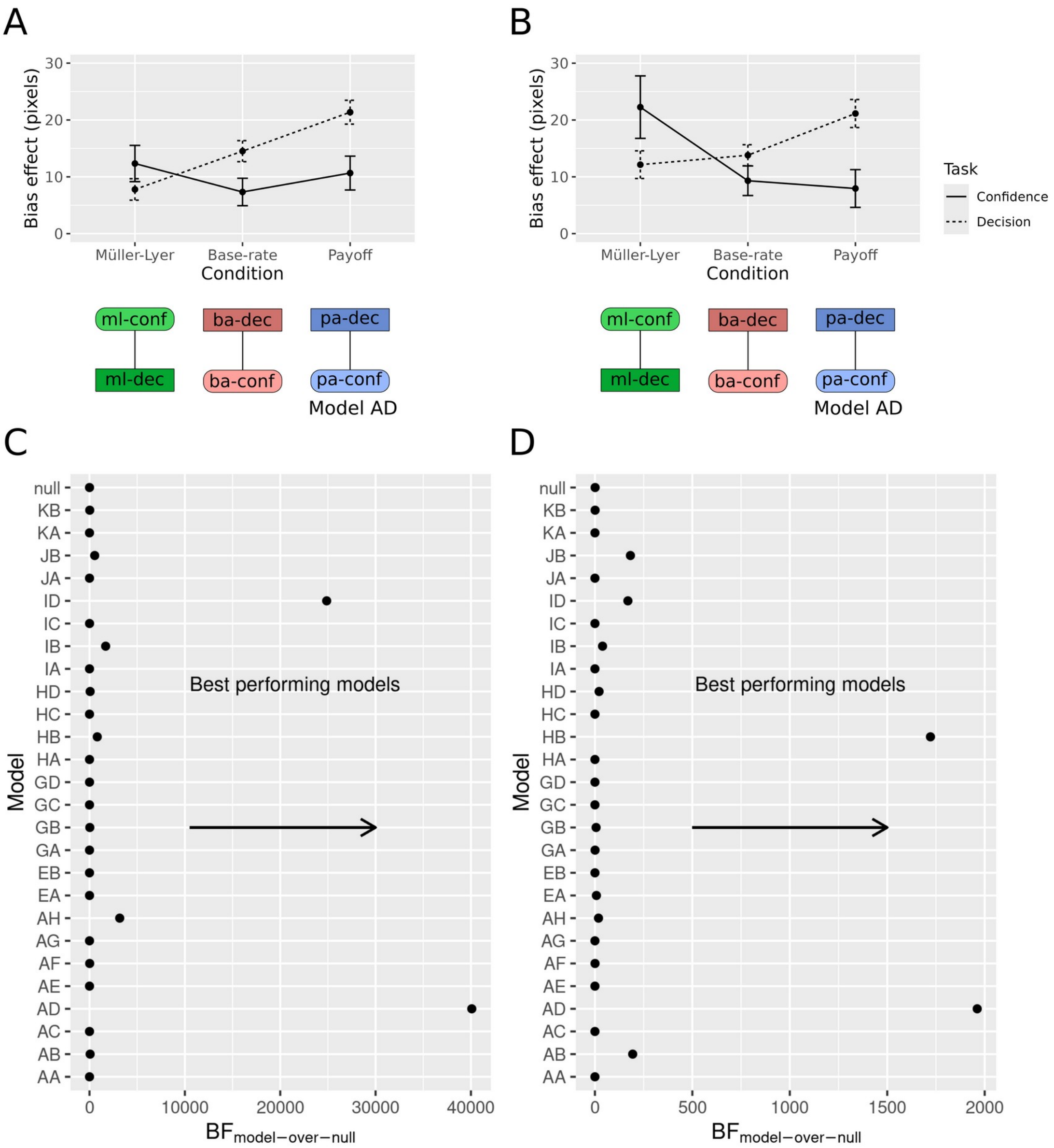

Figure S14: Ordinal modelling of the effects on the decision and confidence task separately for experiment 1 and 2. As in Figure 5, A (experiment 1) and B (experiment 2) indicate the difference in target length (bias-to-long minus bias-to-short) associated with the point of subjective equality (decision task) or the point of maximum uncertainty (confidence task), and the model that best describes the pattern of results (model AD both for experiment 1 and 2). “conf” indicates the bias effect in the confidence task and “dec” indicates the bias effect in the decision task for the Müller-Lyer (ml), base rate (ba) and payoff (pa). Error bars indicate the SEM. C (experiment 1) and B (experiment 2) depicts the BF of each model on the y-axis over the null model. Higher values indicate better performing models. See Supplementary Figure S12 for a graphical depiction of the models tested.

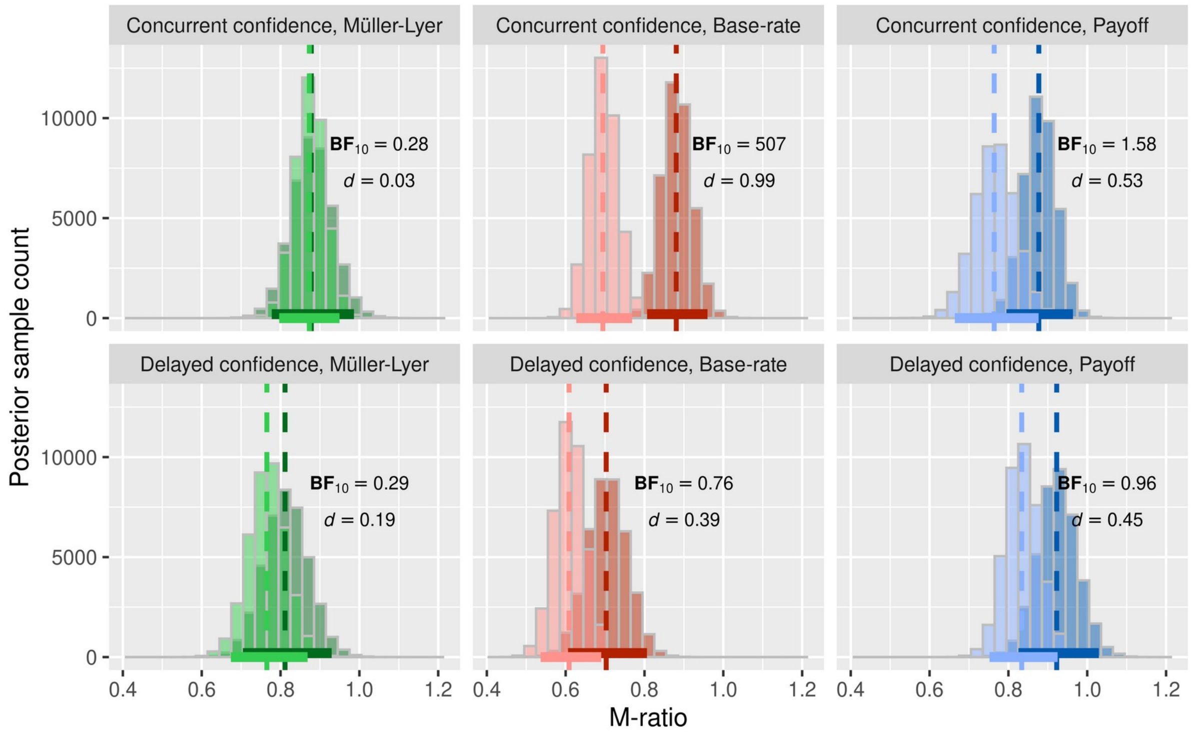

Supplementary figure S15: Experiment 1 and 2 meta-cognitive efficiency (M-ratio). Each distribution depicts the M-ratio posterior distributions for each condition (bias source and bias direction). The top-row panels correspond to experiment 1 (Simultaneous decision and confidence rating) and the bottom-row panels to experiment 2 (Post-decision confidence rating). Vertical dashed lines indicate the mean of the distribution, whereas the thick horizontal line at the base of the distributions depicts the Highest Posterior Density interval (HPDi; 95%). BF were calculated using the Savage-Dickey method using Cauchy distribution with scale 0.707 as a prior. *d* values correspond to Cohen's d effect size coefficients.

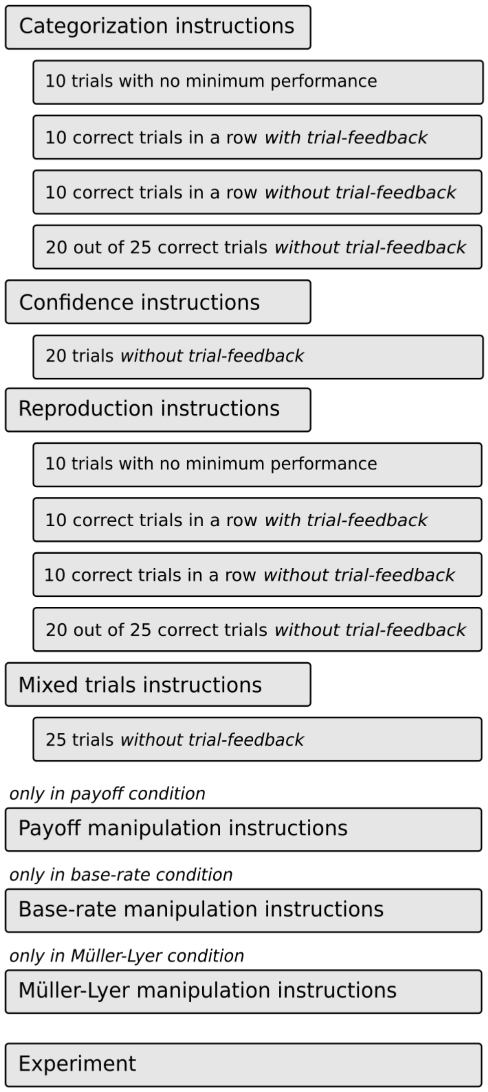

Supplementary figure S16: Experiment general procedure. For each task participants received extensive instructions and completed multiple practice trials. All participants completed the categorization, confidence and reproduction instructions and mixed trials instructions where all tasks were interleaved. Depending on the bias manipulation condition participants received specific instructions related to the manipulation. From top to bottom the figure depicts the order and stages of each part of the instructions. When there was a minimum performance requirement in any of the practice trials sections, participants repeated the practice block until they achieved the expected performance. See General procedure in Methods and Materials for a more detailed description of the procedure.
