# Supplementary material for: Confidence reports during perceptual decision making dissociate from changes in subjective experience
Source: Commun Psychol. 2025 May 21;3:81. doi: 10.1038/s44271-025-00257-y (PMC12095063; doi:10.1038/s44271-025-00257-y)
Supplement: Supplementary file 3 — Reporting Summary [file 44271_2025_257_MOESM3_ESM.pdf]

Corresponding author(s): Nicolas Sanchez-Fuenzalida

Last updated by author(s): Oct 9, 2024

Reporting Summary

Nature Portfolio wishes to improve the reproducibility of the work that we publish. This form provides structure for consistency and transparency in reporting. For further information on Nature Portfolio policies, see our Editorial Policies and the Editorial Policy Checklist.

Statistics

For all statistical analyses, confirm that the following items are present in the figure legend, table legend, main text, or Methods section.

n/a	Confirmed	
☐	☒	The exact sample size (n) for each experimental group/condition, given as a discrete number and unit of measurement
☐	☒	A statement on whether measurements were taken from distinct samples or whether the same sample was measured repeatedly
☐	☒	The statistical test(s) used AND whether they are one- or two-sided<br>*Only common tests should be described solely by name; describe more complex techniques in the Methods section.*
☒	☐	A description of all covariates tested
☒	☐	A description of any assumptions or corrections, such as tests of normality and adjustment for multiple comparisons
☐	☒	A full description of the statistical parameters including central tendency (e.g. means) or other basic estimates (e.g. regression coefficient) AND variation (e.g. standard deviation) or associated estimates of uncertainty (e.g. confidence intervals)
☒	☐	For null hypothesis testing, the test statistic (e.g. F, t, r) with confidence intervals, effect sizes, degrees of freedom and P value noted<br>*Give P values as exact values whenever suitable.*
☐	☒	For Bayesian analysis, information on the choice of priors and Markov chain Monte Carlo settings
☐	☒	For hierarchical and complex designs, identification of the appropriate level for tests and full reporting of outcomes
☐	☒	Estimates of effect sizes (e.g. Cohen's d, Pearson's r), indicating how they were calculated

Our web collection on statistics for biologists contains articles on many of the points above.

Software and code

Policy information about availability of computer code

Data collection	Data was collected using a script made using Python 3.6 and Psychopy (v2020.2.10)
Data analysis	Data analysis was executed using scripts written in R. All data and analysis scripts are available in the repository linked in the next section.

For manuscripts utilizing custom algorithms or software that are central to the research but not yet described in published literature, software must be made available to editors and reviewers. We strongly encourage code deposition in a community repository (e.g. GitHub). See the Nature Portfolio guidelines for submitting code & software for further information.

Data

Policy information about availability of data

All manuscripts must include a data availability statement. This statement should provide the following information, where applicable:

- Accession codes, unique identifiers, or web links for publicly available datasets
- A description of any restrictions on data availability
- For clinical datasets or third party data, please ensure that the statement adheres to our policy

All data is available in https://osf.io/h3uj5/.

Human research participants

Policy information about studies involving human research participants and Sex and Gender in Research.

Reporting on sex and gender	Both sex and gender were not relevant for the current study. Self-reported gender was collected for each experiment merely for descriptive reasons. Only total counts are reported. In experiment 1 there were 29 males and 93 female participants. In experiment 2 there were 19 males and 104 female participants.
Population characteristics	Participants in experiment 1 were 21.3 years old on average (SD=3.3) and 20.5 years old on average (SD=2.8) in experiment 2.
Recruitment	Participants were recruited through an ad published in the University of Amsterdam Laboratory website. The ad was available to anyone registered in the Laboratory website. The pool is composed mostly of undergraduate students. Recruiting undergraduate students is a common practice in experiment psychology research.
Ethics oversight	University of Amsterdam Ethics Review Board (ref. number 2021-BC-14131)

Note that full information on the approval of the study protocol must also be provided in the manuscript.

Field-specific reporting

Please select the one below that is the best fit for your research. If you are not sure, read the appropriate sections before making your selection.

☐ Life sciences ☒ Behavioural & social sciences ☐ Ecological, evolutionary & environmental sciences

For a reference copy of the document with all sections, see nature.com/documents/nr-reporting-summary-flat.pdf

Behavioural & social sciences study design

All studies must disclose on these points even when the disclosure is negative.

Study description	This study consisted of two quantitative experiments with 3 between-subjects bias manipulations (Müller-Lyer, base rate and payoff) and 2 within-subject bias directions (bias-to-short and bias-to-long).
Research sample	The sample consisted in undergraduate students from the University of Amsterdam. 122 participants (29 males, 21.3 years old on average, SD=3.3) and 123 participants (19 males, 20.5 years old on average, SD=2.8) took part in experiment 1 and 2 respectively. Male and female counts refer to self-reported gender.
Sampling strategy	Sampling method: convenience sampling. Since all the confirmatory analyses were conducted in a Bayesian framework, in experiment 1, we collected the data of 30 participants on each of the three bias manipulation conditions (Müller-Lyer, base rate and payoff), removed outliers, and then ran a Bayesian t-test (one-sided, Cauchy prior of 0.707) between the biased to long and short conditions in the decision, confidence and in the reproduction task. If there was moderate evidence for the effect of our manipulation in all tasks (for either the null or the alternative hypothesis), we stopped data collection ($BF10 > 3$ or $BF10 < 0.3$), otherwise we collected five more subjects and repeated the process. In experiment 2 we aimed to collect the same number of participants as in experiment 1, given that both experiments were identical aside from the moment at which participants provided their confidence rating.
Data collection	Data was collected using a computer and a script made using Python and Psychopy. Participants were received by a researcher and then taken to a closed cubicle were they completed the experiment by themselves. Researchers were not blind to the hypothesis nor the study hypothesis during data collection.
Timing	Data collection of Experiment 1 started on the 20 of January 2022 and ended on the 11 of February of the same year. The data collection of Experiment 2 started on the 17 of February 2022 and ended on the 1 of April of the same year.
Data exclusions	The data of a participant was removed if their signal detection theory criterion, d’ or reproduction error fell outside four standard deviations from the sample mean, this is, around the grand average across bias manipulation conditions. Participants with a signal detection theory d’ below zero were also filtered out. In experiment 1 two participants were removed (one reproduction error outlier in the base rate condition and one participant with $d' < 0$ in the Müller-Lyer condition). In experiment 2 four participants were removed (one reproduction error outlier in the base rate condition and three participants with $d' < 0$, two in the payoff condition and one in the Müller-Lyer condition). Participants were also excluded based on curve fit quality (see Analysis – Curve fitting). In experiment 1, eight participants were removed in the Müller-Lyer condition and four in the base rate condition. In experiment 2, ten participants were removed in the Müller-Lyer condition, eleven in the base rate condition and three in the payoff condition.
Non-participation	In Experiment 1, eight participants did not understand the instructions and therefore did not continue to the experiment. Similarly, in Experiment 2, eight participants did not understand the instructions and did not continue to the experiment. Out of the participants that successfully completed the instructions, five participants in Experiment 1 and nine participants in Experiment 2 voluntarily quit the experiment.

Randomization	Participants were allocated to one of the bias manipulations conditions (Müller-Lyer, base rate or payoff) and bias direction (bias-to-long and bias-to-short) as they were recruited. For example, participant 1 was assigned to the Müller-Lyer condition, participant 2 to the base rate condition and so on and so forth. Within each bias condition (Müller-Lyer, base rate or payoff) participants were assigned to start with one bias direction, such that participant 1 in the Müller-Lyer condition started with the bias-to-long condition, whereas the second participant in the Müller-Lyer condition started with the bias-to-short condition.

Reporting for specific materials, systems and methods

We require information from authors about some types of materials, experimental systems and methods used in many studies. Here, indicate whether each material, system or method listed is relevant to your study. If you are not sure if a list item applies to your research, read the appropriate section before selecting a response.

Materials & experimental systems

n/a	Involved in the study
☒	☐ Antibodies
☒	☐ Eukaryotic cell lines
☒	☐ Palaeontology and archaeology
☒	☐ Animals and other organisms
☒	☐ Clinical data
☒	☐ Dual use research of concern

Methods

n/a	Involved in the study
☒	☐ ChIP-seq
☒	☐ Flow cytometry
☒	☐ MRI-based neuroimaging
